# Can green location-oriented policies promote substantive green innovation by enterprises? Evidence from China’s National Eco-Industrial Demonstration Parks

**DOI:** 10.1371/journal.pone.0325666

**Published:** 2025-06-20

**Authors:** Juying Tian, Wei Qiu

**Affiliations:** 1 Economics and Management School, Changji University, Changji, Xinjiang, China; 2 School of Economics, Xinjiang University of Finance and Economics, Urumqi, Xinjiang, China; Southwestern University of Finance and Economics, CHINA

## Abstract

Corporate green innovation is a key practice driving sustainable economic development. Exploring the effectiveness of green location-oriented policies in fostering substantial green innovation among micro-enterprises through a “point-to-area” approach holds practical importance. Drawing on the practice of China’s National Eco-Industrial Demonstration Parks (NEDPs) and utilizing data from A-share listed companies in Shanghai and Shenzhen spanning the period from 2007 to 2022, this paper utilizes a staggered Difference-in-Differences methodology to examine the impact and mechanism of green location-oriented policies on corporate substantial green innovation. The study reveals that the establishment of NEDPs can stimulate substantial green innovation among corporations, with this finding retaining its validity after undergoing a series of robustness test. Analysis of heterogeneity indicates that the incentive effect of NEDPs on corporate substantial green innovation is mainly concentrated in enterprises with low strategic differentiation, low carbon emission performance, and those located in resource-based urban areas. Mechanism analysis indicates that NEDPs jointly promote substantial corporate green innovation mainly through the pressure effects of increased government environmental attention and corporate investment in research and experimental development as well as the incentive effects of green financial support and innovative talent agglomeration. This paper enriches the research on the antecedents of corporate green innovation behavior and provides new micro-level evidence for the effectiveness of green location-oriented policies. It is recommended that governments should con-tenuously strengthen green attention, increase green investment guidance, and cluster innovation factors to effectively promote corporate substantial green innovation.

## 1. Introduction

**Fig 1:**
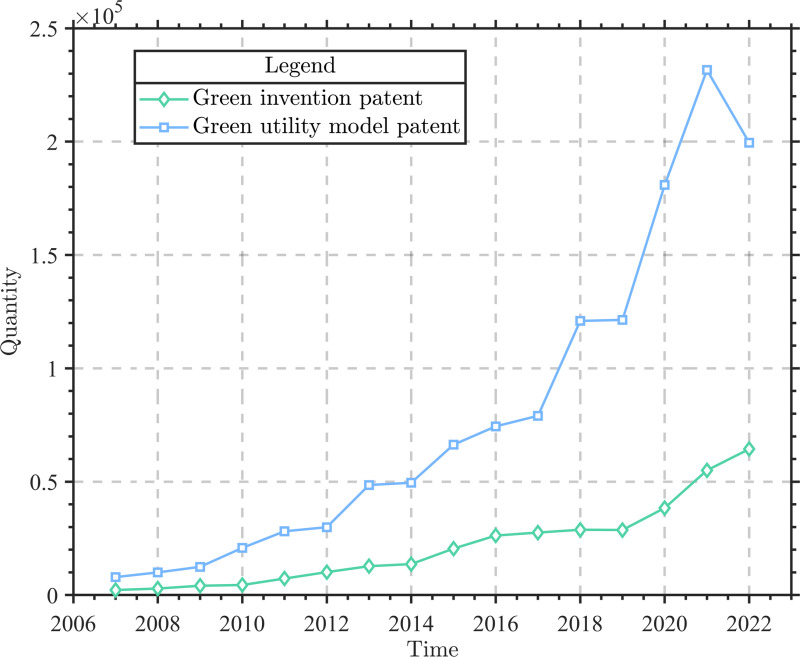
The trend of the number of green invention patents and green utility model patents in China from 2007 to 2022.

Currently, China remains a major global energy consumer and carbon emitter. The issues of resource misallocation and environmental negative externalities caused by the traditional extensive economic model have continued to be prominent, and the contradiction between the rigid demand for energy consumption and ecological protection has become a critical bottleneck constraining China’s sustainable development [1]. As market entities that are both major resource consumers and carriers of technological innovation, enterprises play a pivotal role in promoting green and low-carbon transformation. In recent years, China’s green innovation capability has been significantly enhanced, with the number of green innovation patents increasing from 10,124 in 2007–216,787 in 2022. However, due to challenges such as weak environmental awareness, a lack of innovative talent, and prominent contradictions between short-term profits and long-term investments, enterprises face a situation where the number of strategic green innovations, represented by green utility model patents, is significantly higher than that of substantial technological innovations represented by green invention patents (Fig 1). Substantial green innovation refers to innovative behavior by enterprises that achieves pollution reduction or resource efficiency improvement through core technology breakthroughs and generates real environmental benefits [2]. It plays a crucial role in accelerating the achievement of China’s “dual carbon” goals. In contrast, strategic green technological innovation represents short-term, low-investment measures taken by enterprises to respond to regulatory pressures or market demands, which cannot truly achieve green and sustainable development. Especially in the context of slowing macroeconomic growth, the survival environment for enterprises has worsened, and they face greater difficulties in improving the quality and efficiency of green innovation. Therefore, investigating methods to enhance enterprises’ role as innovation leaders and facilitate the development and implementation of substantial green and low-carbon technologies hold practical importance.

Since the reform and opening-up, urban industrial parks have been the main driving force for promoting the development of Chinese enterprises. However, traditional industrial parks are a “double-edged sword.” They commonly adopt a park development model characterized by “low value, high energy consumption, and heavy pollution,” which can easily lead to problems such as inadequate resource utilization, weak innovation momentum, and environmental pollution. As the economic development model shifts towards synergistic efficiency in terms of reducing pollution and cutting carbon emissions reduction, China has taken the lead in initiating ecological transformation activities within industrial parks since 2001. By establishing China’s National Eco-Industrial Demonstration Parks (NEDPs), promoting industrial agglomeration and circular economy development has become a crucial pathway to address the environmental negative externalities of traditional parks, enhance the flow of enterprise factors, and promote green development among enterprises. As a green location-oriented policy, on the one hand, NEDPs enforce strict environmental regulations and provide special funding support to guide enterprises within the parks towards low-carbon emissions [3]. On the other hand, they inevitably influence the innovative activities of enterprises outside the parks in the city through policy spillovers, supply chain reconstruction, changes in factor prices, and the flow and exchange of resources and information.

So, faced with the dual-effect policy of pressure and incentive represented by the NEDPs, how will local enterprises make their choices? Will they lean towards substantive engage in green innovation or adopt strategic approaches to green innovation? Furthermore, do enterprises in different regions or with different characteristics lead to heterogeneity in the impact of NEDPs on their substantive green innovation? What are the mechanisms behind these impacts? The in-depth exploration of the above issues aims to reveal the motivations behind enterprises’ choices for substantive green innovation, and to explore a concrete and feasible path for the government to devise more targeted and efficient green location-oriented policies and regulate enterprises’ green innovation behaviors.

## 2. Literature review

Literature closely related to the theme of this paper can be mainly divided into two categories. The first category focuses on the green innovation effects of Eco-Industrial Parks (EIPs). Early eco-industrial parks, exemplified by the Kalundborg Industrial Symbiosis in Denmark, were centered around the concept of a green circular system [4] China also launched the NEDPs in 2001, as a green place-based policy aimed at minimizing the negative environmental impacts of local industrial development [5] Numerous studies have investigated the green innovation effects of the NEDPs at the regional level. Cao et al. found that this policy can effectively reduce emissions of sulfur, nitrogen oxides, and carbon dioxide [6]. The policy’s role in promoting regional innovation is considered an important pathway for generating green effects [7]. Liu et al. (2023) argued that the establishment of NEDPs can directly enhance regional innovation capabilities, drive research into advanced production technologies, and thereby reduce carbon emissions [8]. Other viewpoints suggest that the establishment of NEDPs contributes to improving the local innovation environment and optimizing energy efficiency [9]. Specifically, this policy promotes the spatial agglomeration of high-quality human capital and green enterprises, increasing local green innovation activities [10]. It further enhances knowledge spillovers through eco-industrial clusters, fostering regional green technological innovation [11]. Luo et al. (2024) similarly believed that NEDPs facilitate the dissemination of environmental protection technologies and management knowledge across the city, thereby elevating the overall level of green innovation and driving economic development [12]. Other pathways include the findings of Song and Zhou (2021), who pointed out that NEDPs improve energy efficiency from the perspective of innovation compensation by providing targeted green innovation subsidies [13], as well as Liu et al. (2021), who argued that these parks drive technological innovation and generate green effects through measures such as increasing financial investment, offering tax incentives, and encouraging the mobility of high-quality talent [14].

The second category of literature relevant to this paper focuses on the impact of policies on green technological innovation. Some studies have examined policies that can incentivize enterprise innovation, finding differences in policy effectiveness. Kong et al., based on a study of the national innovation-driven pilot policy, discovered that this policy significantly promotes green technological innovation in enterprises [15]. Li et al. investigated government subsidies in China’s new energy vehicle industry and found a U-shaped relationship between government subsidies and corporate green innovation, indicating better policy effectiveness after overcoming initial information asymmetry [16]. Substantive green technological innovation is oriented towards environmental protection and features technological progress, yet the innovation process is fraught with high risks and uncertainties [17]. Consequently, Liang et al. (2025) [18] found that while the designation of high-tech enterprises can promote corporate green innovation, enterprises often exploit investors’ lack of information, favoring strategic innovation, thereby generating innovation patent bubbles. Another category of research focuses on policies that exert pressure effects on enterprise innovation. Chen et al. classified institutional pressures into coercive pressure and normative pressure, finding that both can effectively drive enterprises to participate in green innovation [19]. Among these, environmental penalties, such as coercive pressure, can significantly promote substantive green technological innovation in enterprises through pressure effects [20]. regarding normative pressure, PU and Ouyang et al. (2023) [21] found that the carbon emissions trading system can also effectively drive green technological innovation in enterprises. However, numerous economic perspectives argue that institutional pressures increase enterprise costs and hinder effective investment in green innovation [22]. Due to the existence of the green innovation paradox, policies aimed at reducing resource consumption and pollution often yield contradictory results in corporate practice. For example, Liu and Dong (2022) [23].found that green credit policies fail to drive substantive innovation in heavily polluting enterprises, possibly due to the insufficient innovation resources of these enterprises themselves Bi et al. (2024) [24] also found that under the pressure of the new Environmental Protection Law, green innovation in heavily polluting enterprises is mostly non-substantive, while pilot projects integrating technology and finance can significantly promote corporate green innovation [25].

Existing research has provided theoretical support for how NEDPs promote green technological innovation, yet there are still many areas needing expansion. On the one hand, current research has only examined the impact of NEDPs on green technological innovation from the meso-level perspective of cities. However, from a practical standpoint, enterprises are the primary agents of innovation, and the interactive relationship between micro-level enterprise characteristics and meso-level institutional environments remains unclear. The underlying logic and empirical evidence still require deeper research for supplementation. On the other hand, although existing studies have elaborated on how to promote green innovation from aspects such as talent aggregation, financial subsidies, and increasing R&D investment, most of these studies are still confined to qualitative analysis. There is a notable deficiency in quantitative research when it comes to in-depth analysis of how NEDPs affect substantive green innovation in enterprises, especially regarding their mechanisms of action.

In view of this, this paper makes the following contributions: Firstly, existing research primarily relies on regional-level data, while this paper uses more micro-level enterprise data to identify the promotional effect of NEDPs on substantive green innovation by enterprises, thereby broadening the research scope of existing literature. Secondly, this paper further distinguishes green technological innovation into substantive green innovation and strategic green innovation, providing a more comprehensive perspective for understanding the impact of green location-oriented policies on corporate green innovation and deepening the research depth of existing literature. Finally, in terms of the theoretical framework, this paper constructs a theoretical framework that integrates the dual effects of pressure and incentives from both governments and enterprises, systematically exploring the theoretical mechanism through which NEDPs drive substantive green innovation by enterprises and identifying the main points of focus and specific channels for promoting substantive green innovation by enterprises.

The subsequent structure of this paper is as follows: Section 3 analyzes the relevant mechanisms and proposes hypotheses. Section 4 introduces the methods and data sources used in this paper. Section 5 presents empirical results on how the establishment of NEDPs affects substantive green innovation by enterprises. Finally, Section 6 elaborates on the conclusions and policy recommendations.

## 3. Research hypotheses

Green innovation is influenced both by internal drivers and in response to external policy environments [2]. For NEDPs, as targeted policies with problem-oriented characteristics, they can impact on substantial green innovation among enterprises both inside and outside the park through internal drivers and external responses.

Firstly, the pressure effect of enhancing governmental focus on environmental protection. Theoretically, according to the “Porter Hypothesis,” the tightening of environmental regulations can elevate the level of technological innovation within enterprises [26]. NEDPs possess clear construction standards, necessitating local governments to conduct quantitative assessments of core indicators such as stringent environmental benchmarks, energy consumption elasticity coefficients, and renewable energy proportions to guide the establishment of eco-industrial parks. Furthermore, every three years, already approved NEDPs undergo reviews based on these standards, thereby establishing mandatory norms [27]. This system enhances local governments’ attention to environmental protection. On one hand, this heightened governmental focus on environmental protection forces enterprises within the parks to continuously engage in cleaner and more circular transformations of by-products, waste materials, or production technologies and equipment. Consequently, this propels enterprises to make substantive green technological innovations, thereby improving the material and energy exchange efficiency among enterprises within the parks. On the other hand, according to the government attention theory, for enterprises outside the parks, when the focus of governmental environmental protection efforts becomes clearer, enterprises will proactively strengthen their environmental awareness and actively respond to governmental environmental policies to secure more resource support and meet the requirements for green procurement by the government [28]. To forge a positive green corporate image, enterprises shift their operational models from short-term pollution control to long-term pollution prevention. By engaging in continuous substantive innovative activities, they shape their unique green image in pursuit of sustained competitive advantages within the local supply chain market.

In view of this, the following hypothesis is proposed:

Hypothesis 1: NEDPs promote substantive green innovation in enterprises by enhancing government attention to environmental protection.

Secondly, the pressure effect of increasing enterprises’ R&D investment. When the benefits of increasing R&D investment surpass the exit costs, the NEDPs policy triggers an “innovation compensation effect,” continuously enhancing enterprises’ motivation for substantive green innovation. The theory of New Institutional Economics points out that to survive in a specific environment, an organization must comply with various norms of that environment to gain recognition and support from stakeholders, thereby obtaining the corresponding “legitimacy” qualification and maintaining its “legitimate” status [29]. For enterprises within the parks, to meet the assessment requirements of the NEDPs, they face a choice between exiting polluted industries or increasing R&D investment and actively adopting substantive green innovations to satisfy the NEDPs’ criteria. Enterprises exiting polluted industries to enter new sectors will face substantial cost pressures, including the loss of existing assets, equipment, facilities, and customer bases, as well as bearing the costs of market development to enter new industries [30]. Therefore, only a few enterprises will choose to exit polluted industries, while most will increase their R&D investment to achieve substantive green innovation to meet compliance requirements. Furthermore, compared to traditional industrial parks, NEDPs establish a green industrial chain [14] through the complementarity of product and business modules, realizing reunification across the entire industrial chain process from design, production, sales, waste disposal, and recycling. The knowledge spillover within these parks exhibits a green bias. Hence, for enterprises outside the parks, on the one hand, industrial collaborative innovation projects led by core enterprises within the parks prompt external enterprises to increase their investment in joint R&D. On the other hand, enterprises within eco-industrial parks achieve green technology spillovers through material circulation networks [31], compelling related enterprises to increase their R&D investment to synchronize process upgrades.

In view of this, the following hypothesis is proposed:

Hypothesis 2: NEDPs promote substantial green innovation in enterprises by increasing their R&D investment.

Thirdly, the incentive effect of government green financial support. According to the resource-based view, a firm’s capabilities stem from the resources it can access [32], and governmental green fiscal support will augment the resources available to firms for substantive green innovation investment. On the one hand, NEDPs must meet stringent environmental standards. To satisfy assessment requirements, governments need to expand green investments [33] and incentivize enterprises to purchase environmental protection equipment through a series of economic measures such as tax incentives, green credit, and subsidies, thereby promoting the application and promotion of substantive green innovation technologies among enterprises within the parks. On the other hand, to continuously enhance the economic and ecological advantages of eco-industrial parks, governments will increase green investments and prioritize the allocation of production factors to low-carbon industries. By offering incentives such as capital support, they attract related green industries to locate within the parks [34], which is conducive to expanding the scope of entities engaged in substantive green innovation. Additionally, from a procurement perspective, government departments can serve as “lead users” by clarifying market demand information for products and services [35]. Therefore, government green procurement can leverage the “leverage” effect of financial funds to incentivize enterprises to introduce green processes and iteratively update green products, thereby expanding the local green market. Guided by the “pull” of government demand, it reduces market uncertainty for local enterprises to engage in substantive green technological innovation [36]. Furthermore, government green investments will facilitate the construction of infrastructure such as public service platforms for green industries, generating a scale effect for substantive green technological innovation.

In view of this, the following hypothesis is proposed:

Hypothesis 3: NEDPs promote substantial green innovation in enterprises by expanding green financial support.

Fourthly, the incentive effect of agglomerating innovative talents. Modern economic growth theory indicates that talent is the primary driving force for sustained regional economic growth and the realization of economic progress [37]. Production factors such as labor tend to flow after comparing regional benefits, and the continuous aggregation of talent can provide essential conditions for enterprises to conduct substantive green technology innovation research, as well as for the transformation and application of research outcomes. On the one hand, when choosing a place of residence, innovative talents demonstrate a significant “voting with their feet” effect concerning environmental quality, tending to prefer areas with better environments [38]. Urban pollution often has a repelling effect on innovative human capital. However, NEDPs, with their clear construction standards and stringent environmental requirements, effectively promote carbon sequestration, oxygen release, pollution resistance, and biodiversity conservation in cities, playing a significant role in purifying and restoring the urban ecological environment, thereby attracting many innovative talents. On the other hand, studies have shown that the greater the diversity of enterprise types within a city’s industrial chain, the more favorable it is for acquiring high-value green factors [39]. The construction of NEDPs promotes the transformation of urban industries towards emerging, modern, and high-end industries, attracting talents from surrounding areas to gather in the demonstration parks, providing diversified talent support for technological innovation within the parks [40]. In addition, NEDPs are usually equipped with a complete innovative ecosystem and platforms, providing abundant R&D resources and broad entrepreneurial opportunities for innovative human capital. At the same time, enterprises within the parks have established close cooperative relationships with academic institutions and research organizations in the city, promoting the sharing of green knowledge through clean technology exchanges and cooperation, and successfully achieving substantial green innovation transformation.

In view of this, the following hypothesis is proposed:

Hypothesis 4: NEDPs promote substantial green innovation in enterprises by aggregating innovative talent.

Hypothesis 5: NEDPs can promote substantial green innovation in enterprises.

**Fig 2:**
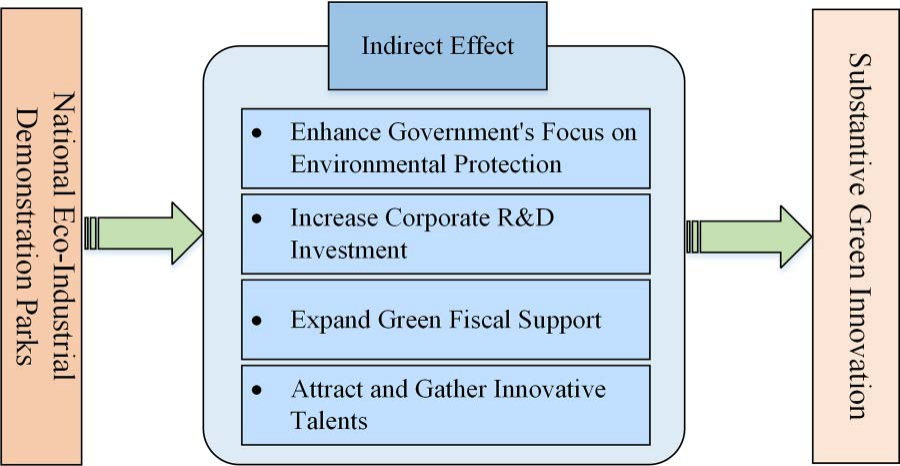
The process by which NEDPs influences substantive green innovation.

The influence mechanism is illustrated in Fig 2.

## 4. Materials and methods

### 4.1. Data sources

Considering the new accounting standards issued in China in 2006, which improved the comparability of financial information starting from 2007, this study selects data from Chinese A-share listed companies spanning from 2007 to 2022 as the research sample. The CSMAR database is utilized as the source for basic corporate information and financial data. Corporate patent application data are obtained from the China Research Data Services Platform (CNRDS). City-level control variables are derived from various years of the “China Statistical Yearbook”. The general process for matching these databases is as follows: (1) Company samples with ST labels and missing key variables in the CSMAR database are excluded; (2) Based on China’s city codes, listed company data is manually matched in the cities where the companies are headquartered; (3) Winsorization is applied to the main variables at the 1% level. After screening, the final merged dataset comprises 27,690 city-firm-year observations across 235 cities and 3,499 listed companies.

### 4.2. Measurement of key variables

#### 4.2.1. National demonstration eco-industrial parks.

If the city *i* has a national eco-industrial park pilot project at time *t*, it is deemed to possess a National Demonstration Eco-industrial Parks and is given a value of 1. Conversely, if it does not, it is given a value of 0.

#### 4.2.2. Substantial green innovation.

On the one hand, the granting of patents involves a lengthy process including application, acceptance, examination, and others, which can take considerable time. During the stage of green patent application, the technological innovations are likely to have already influenced the production and operational activities of enterprises. Hence, in comparison to the number of green patents awarded, the number of green patent applications submitted is considered more reliable and timelier, better reflecting the innovative strategic choices of enterprises when facing policy adjustments. On the other hand, green invention patents are relatively the most challenging to develop and hold the highest market worth. Thus, drawing inspiration from the approach of existing literature, this paper utilizes the total number of green invention patent applications in a given year to measure corporate substantial green innovation, while the total number of green utility model patent applications is used to gauge corporate strategic green innovation [41].

#### 4.2.3. control variable.

To reduce the influence of unobserved variables on the outcomes, this paper adopts the methodology proposed in previous literature [42]. It selects a range of variables that could potentially influence corporate substantial green innovation. On the enterprise level, these include the debt-to-asset ratio (LEV), which directly influences corporate financial stress and, consequently, substantial green innovation, and is quantified as the year-end ratio of total liabilities to total assets [43]. Additionally, the book-to-market ratio (BM), serving as an indicator of market recognition and influencing corporate innovation decisions, is calculated as the ratio of book value to total market value [44]. The tangible asset ratio (TANG) is represented by the proportion of the sum of net fixed assets and inventories to the total assets [45]. Furthermore, the top ten shareholder ownership ratio (TOP) is utilized, reflecting the shareholding of the top ten shareholders [46]. The percentage of independent directors (IND) is measured by the proportion of independent directors out of the total share capital to the overall count of board members [47]. Lastly, the quick ratio (QR), indicating liquidity, is computed as the sum of cash, short-term investments, notes receivable, and accounts receivable to current liabilities [48]. At the city level, the paper considers the industrial structure (THI) [49], gauged by the ratio of the GDP of the tertiary industry to the total GDP of the city. Additionally, the registered population (POP) of the city is considered a demographic factor [50].

### 4.3. Model construction

This paper posits that the establishment of the NEDPs can be regarded as a quasi-natural experiment. Since the pilot cities of NEDPs are selected in multiple batches and not in the same year, this paper employs the Staggered Difference-in-Differences. The rationale is that this model can effectively address the complex data relationships arising from the gradual implementation of policies or interventions across different time periods and regions, and, after correcting for heterogeneous treatment effects, it can assess the impact of NEDPs on enterprises’ substantive green technology innovation with relatively high accuracy. The specific design is outlined as follows:


$$GTI_{i,t}=\alpha_{0}+\alpha_{1}NEDP_{i,l,t}+\sum_{m}\alpha_{m}Controls_{i,l,t}+\lambda_{t}+\mu_{i}+\gamma_{l}+\varepsilon_{i,l,t}$$ (1)

In formula (1), *i* and *t* represent the enterprise and the corresponding year, respectively. *NEDP* denotes National Demonstration Eco-industrial Parks, *GTI* signifies the enterprise’s green innovation output, and *Controls*_*i,t*_ is a set of control variables. *λ*_*t*_, *μ*_*j*_ and *γ*_*l*_ indicate the yearly fixed effects, firm and city, respectively, while *ε*_*i,t*_ represents the fixed effects as well as the random error components.

Table 1 displays the definitions of the key variables along with the descriptive statistics of the raw data.

**Table 1:** Descriptive statistics.

Type of variable	Variable	Mean	S. D	Minimum	Maximum
**Dependent Variable**	GTI	2.861	17.232	0.000	809.000
**Explanatory Variable**	NEDP	0.320	0.466	0.000	1.000
**Firm-level Control Variables**	LEV	0.402	0.200	0.008	1.687
	BM	0.354	0.160	−0.347	1.290
	TANG	0.930	0.089	0.148	1.000
	TOP	59.689	15.293	8.970	101.160
	IND	37.451	5.565	0.000	80.000
	QR	2.376	3.965	0.038	179.578
**Regional-level Control Variables**	THI	55.091	13.533	11.800	83.860
	POP	790.438	509.485	18.140	3416.000

## 5. Empirical results

### 5.1. Benchmark regression model

Prior to performing the basic regression analysis, this paper utilized the variance inflation factor (VIF) method to examine multicollinearity among the variables. The results showed that both the overall VIF value for the main model and the individual VIF values for the explanatory variables were below 4. Specifically, the highest VIF value was 1.68, the lowest was 1.00, and the average VIF value was 1.25. These results suggest that there is no significant issue of multicollinearity among the variables.

Table 2 presents the benchmark regression results for the impact of NEDPs on firms’ substantive green innovation. The results indicate that, without including control variables, the estimated coefficient of NEDP in column (1), the coefficient is significantly positive at the 5% level. To enhance the accuracy of the conclusions, control variables are subsequently included, and the estimated coefficient of NEDP in column (2) becomes significantly positive at the 1% level, suggesting that Hypothesis 5 is tentatively supported. The estimated coefficients for NEDP in both columns (3) and (4) are statistically insignificant, indicating that NEDPs do not promote firms’ strategic green innovation. The possible reasons why NEDPs can only drive substantive green innovation among firms are as follows: Firstly, based on the Government Attention Theory, NEDPs elevate governmental focus on environmental protection through clear standards and regular reviews, compelling firms to adopt cleaning and recycling practices, thereby enhancing their environmental awareness and green innovation capabilities. Secondly, New Institutional Economics posits that organizations must conform to environmental norms to gain stakeholder recognition. Stringent assessments drive firms to increase R&D investments for compliance through green innovation, while park-based industrial chains and knowledge spillovers encourage external firms to collaborate on R&D. Thirdly, Resource-Based View asserts that firms leverage resources such as green fiscal support and green procurement to reduce market risks, expand green markets, and drive technological innovation. Finally, the “Voting with One’s Feet” Theory suggests individuals migrate based on regional environmental and institutional differences. A park with a strong ecosystem and diversified industrial chain attracts talent, offering multifaceted support, abundant R&D resources, and facilitating green knowledge sharing and substantive innovation transformation. As per the analysis of control variables, factors including LEV, BM, TOP, QR, THI, POP have all exhibited significant positive impacts on firms’ engagement in substantive green innovation. These indicators collectively reflect the financial robustness, market recognition, short-term liquidity, industry trends, and regional market potential of enterprises, all of which foster their willingness to innovate and allocate resources toward this end. Conversely, a higher ratio of tangible assets may indicate a reliance on traditional assets and models, thereby hindering the transition toward substantive green innovation among enterprises.

**Table 2:** Benchmark regression results.

Variable	(1)	(2)	(3)	(4)
**NEDP**	0.078^**^	0.079^***^	0.044	0.043
	(0.030)	(0.030)	(0.030)	(0.030)
**LEV**		0.312^***^		0.327^***^
		(0.065)		(0.066)
**BM**		0.157^***^		0.176^***^
		(0.054)		(0.052)
**TANG**		−0.172^*^		−0.194^**^
		(0.093)		(0.092)
**TOP**		0.002^*^		0.001
		(0.001)		(0.001)
**IND**		0.001		−0.000
		(0.001)		(0.002)
**QR**		0.003^**^		0.003^**^
		(0.001)		(0.001)
**THI**		0.004^*^		0.001
		(0.002)		(0.002)
**POP**		0.138^*^		0.046
		(0.081)		(0.081)
**Individual fixation**	YES	YES	YES	YES
**Time fixation**	YES	YES	YES	YES
**City fixation**	YES	YES	YES	YES
**_cons**	0.454^***^	−0.799	0.474^***^	0.034
	(0.010)	(0.567)	(0.010)	(0.569)
**N**	27690	27690	27690	27690
**R** ^**2**^	0.662	0.663	0.660	0.661

**Note:** 1)

* p<0.1,

** p<0.05,

*** p<0.01, are significant at 10%, 5%, and 1% confidence levels, respectively. 2) Robust standard errors, which are clustered at the enterprise level, are shown in parentheses.

### 5.2. Parallel trend test

The credibility of the Difference-in-Differences method hinges critically on the parallel trend’s assumption between the treatment and control groups. To verify this assumption and examine the temporal variation in policy effects, this paper draws on the event study methodology to construct a corresponding analysis model. The specific design is outlined as follows:


$$\begin{array}{c} \begin{array}{c} GTI_{i,t}=\alpha_{0}+\alpha_{1}NEDP_{i,l,t}^{-9}+\alpha_{2}NEDP_{i,l,t}^{-8}+\cdots +\alpha_{18}NEDP_{i,l,t}^{9}\\ +Controls_{i,l,t}+\lambda_{t}+\mu_{i}+\gamma_{l}+\varepsilon_{i,l,t} \end{array} \end{array}$$ (2)

*NEDP*_*i,l,t*_^*-q*^ represents an indicator that is set to 1 if city *i* in location l is in the *q* year prior to its first establishment of a NEDPs, and 0 otherwise. *NEDP*_*i,l,t*_^*q*^ denotes an indicator that takes a value of 1 if city *i* in location *l* is in the *q* year after its initial establishment of NEDPs, and 0 otherwise. The other variables and specifications remain the same as in Model (1).

**Fig 3:**
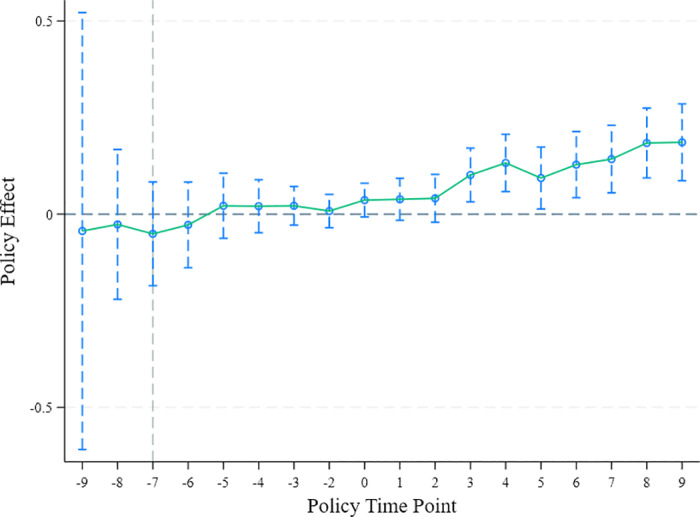
Trend comparison chart for parallel groups.

In this paper, the year directly before the implementation of policy is taken as the baseline time point (period −1). Observing Fig 3, it is evident that the relevant indicators do not demonstrate statistical significance immediately before the policy implementation initiation, suggesting that the parallel trends assumption in Model (1) is largely valid, thereby enhancing the credibility of the baseline analysis. Next, we turn to explore the temporal evolution of the policy effects. Fig 3 intuitively demonstrates that, when compared to the control group, the treatment group exhibits a significantly increasing trend in the frequency of investment activities in the years following the creation of the national eco-industrial showcase park. This positive effect reaches statistical significance in most years. The data in Fig 3 not only provides empirical support for the conclusions in Table 2 but also reveals the time-lagged nature of the co-construction park effect. This lagged effect may be attributed to the fact that we define the policy initiation point as the establishment or approval of the park. The process from parking planning and construction, infrastructure improvement, to attracting investment and promoting substantial green transformation of enterprises requires time accumulation. Therefore, the policy effects manifest as a lagged response in the charts.

**Fig 4:**
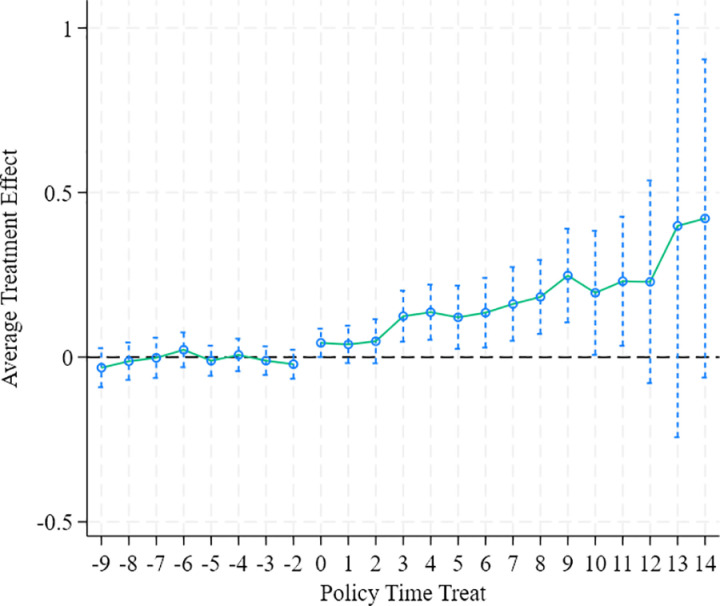
CSdid.

In the staggered Difference-in-Differences (DID) model, the estimation of two-way fixed effects may suffer from bias issues. To address this problem, this study adopts the staggered doubly robust estimator proposed by Callaway and Sant’Anna [51] for robustness verification. Rooted in the principle of doubly robustness, this method can effectively mitigate biases in DID estimation. The core idea is to subdivide the sample into different subgroups, evaluate the treatment effect for each subgroup separately, and then integrate these effects using a specific method to calculate the Average Treatment Effect on the Treated (ATT) over the sample period. The integration strategy emphasizes reducing the weights of potentially biased subgroups. Detailed results are presented in Table 3 and Fig 4. The findings indicate that all four types of average treatment effects consistently show that NEDPs can significantly promote substantial green innovation among enterprises, which is consistent with the baseline results, further validating the robustness of the conclusions.

**Table 3:** Endogenous test results.

Variable	(1)	(2)	(3)	(4)
**ATT**	0.122^***^			
	(0.033)			
**Pre_avg**		−0.008		
		(0.008)		
**Post_avg**		0.181^***^		
		(0.056)		
**CAverage**			0.073^**^	
			(0.030)	
**GAverage**				0.118^***^
				(0.032)

**Note:** 1)

* p<0.1,

** p<0.05,

*** p<0.01, are significant at 10%, 5%, and 1% confidence levels, respectively. 2) Robust standard errors are in parentheses, clustered at enterprise level.

### 5.3. Robustness test

The previously presented empirical findings suggest that NEDPs has a positive and significant impact on green innovation, and these results remain consistent even after addressing potential endogeneity concerns. To reinforce the credibility of these conclusions, this paper will undertake a range of robustness tests.

#### 5.3.1. Exclude municipalities directly administered by the central government and provincial capitals.

Cities directly under the central government’s jurisdiction and provincial capitals often possess substantial economic scales and high-level corporate R&D capabilities. The economic development levels and industrial structures of these cities exhibit notable differences compared to those of other non-provincial capital cities. Considering this, the present study excludes to reduce potential biases due to sample selection, this study includes cities directly under the central government’s administration and provincial capitals. The results presented in Column (1) of Table 4 indicate that the estimated coefficient of NEDP is significantly positive at the 1% level, aligning with the baseline regression outcomes.

**Table 4:** Robustness test results.

Variable	(1)	(2)	(3)	(4)	(5)	(6)	(7)
**NEDP**	0.082^***^	0.073^**^	0.079^***^	0.078^**^	0.086^**^	0.071^**^	0.065^**^
	(0.030)	(0.030)	(0.030)	(0.030)	(0.036)	(0.033)	(0.030)
**EMP**		−0.000^***^					
		(0.000)					
**Control variable**	YES	YES	YES	YES	YES	YES	YES
**Industry fixation**	YES	YES	YES	YES	YES	YES	YES
**Time fixation**	YES	YES	YES	YES	YES	YES	YES
**City fixation**	YES	YES	YES	YES	YES	YES	YES
**Province fixation**	NO	NO	YES	NO	NO	NO	NO
**_cons**	−0.829	−0.686	−0.799	−0.960	−1.251^**^	−1.551^***^	−1.069^**^
	(0.566)	(0.467)	(0.567)	(0.616)	(0.609)	(0.552)	(0.497)
**N**	27689	27659	27690	22598	13124	18877	22,011
**R** ^**2**^	0.663	0.660	0.663	0.674	0.601	0.607	0.608

**Note:** 1)

* p<0.1,

** p<0.05,

*** p<0.01, are significant at 10%, 5%, and 1% confidence levels, respectively. 2) Robust standard errors, which are clustered at the enterprise level, are shown in parentheses.

#### 5.3.2. Including extra control factors.

High labor intensity may pose managerial challenges, such as coordinating employee collaboration and ensuring the equitable allocation of innovative resources. The heightened complexity in management may subsequently diminish innovation efficiency and the quality of innovative outputs. The results presented in Column (2) of Table 4 reveal that after incorporating EMP as an additional control variable, the estimated coefficient of NEDP remains positive at the 5% significance level, indicating that the baseline regression results retain their robustness.

#### 5.3.3. Adding fixed effects.

In this study, we have further incorporated provincial fixed effects to account for unobservable, non-time-varying characteristics of local enterprises that may be influenced by provincial and macroeconomic factors. The regression outcomes displayed in column (3) of Table 4 reveal that the coefficient for NEDP is statistically significant at the 1% level, reinforcing the robustness of our research findings.

#### 5.3.4. Narrowing the sample interval.

The timing of sample selection may introduce bias into the test results, prompting the exclusion of sample data from the first and last years. The results in column (4) of Table 4 show that the estimated coefficient for NEDP is significantly positive at the 5% level, once again illustrating the robustness of the baseline regression results presented in this paper.

#### 5.3.5. PSM-DID.

Due to potential selective bias arising from systematic differences in national eco-industrial demonstration zones, endogeneity issues stemming from such bias need to be mitigated. To this end, we conduct robustness checks using the Propensity Score Matching-Difference in Differences (PSM-DID) method. Based on the control variables, we employ the nearest neighbor matching with ratios of 1:1, 1:2, and 1:3 for the samples, and the matching results pass the balance test. Subsequently, we re-run the regression model (1) using the PSM-matched samples, with the results presented in Table 4, columns (5) to (7). Columns (1) to (3) correspond to the test results for nearest neighbor matching with ratios of 1:1, 1:2, and 1:3, respectively. According to these results, there is a significant positive correlation between national eco-industrial demonstration zones and substantial green innovation by enterprises, supporting the original conclusions of this paper.

#### 5.3.6. Placebo test.

**Fig 5:**
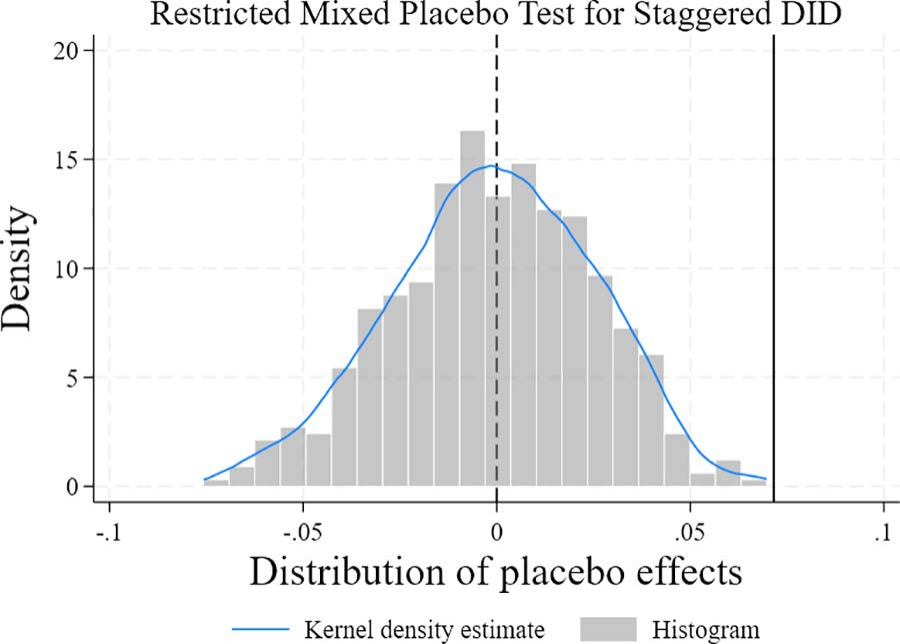
Placebo test.

To avoid sample selection bias, a placebo test was conducted by randomly selecting policy implementation years and a corresponding number of cities to replace the existing treatment group, with the process repeated 500 times to overcome potential sample bias issues. The results of the placebo test are shown in Fig 5. The vertical solid line represents the true coefficient, and many causal regression results are insignificant and cluster around 0, passing the placebo test.

### 5.4. Heterogeneity analysis

To offer more comprehensive empirical evidence, this paper delves into the heterogeneity of the impact of NEDPs on firms’ substantive green innovation, considering factors such as firms’ strategic deviation, carbon emission performance, and whether they are in resource-based cities.

#### 5.4.1. Heterogeneity in strategic deviation.

The results in column (1) of Table 5 indicate that for listed companies with lower strategic deviation, the estimated coefficient of NEDP is significantly positive at the 1% level. In contrast, the estimated coefficient of NEDP in column (2) of Table 5 is not significant. This may be attributed to the fact that firms with lower strategic deviation are generally more inclined to adhere to established market rules and industry standards, adopting relatively conservative and stable business strategies [52]. When confronted with changes in the external environment, such firms tend to prefer incremental innovation within the existing framework rather than disruptive changes. However, NEDPs create a relatively stable environment conducive to green innovation for these firms by providing stringent regulatory measures, preferential tax policies, and effective voluntary advocacy. These firms can more easily adapt to the policy orientation of the parks, leveraging their resources and support to conduct R&D and application of green technologies, thereby enhancing their green innovation capabilities. In contrast, firms with higher strategic deviation are typically more inclined to adopt differentiated market strategies and innovative business models. They may focus more on exploring new markets, developing new technologies, or providing new service methods to distinguish themselves in the fiercely competitive market. However, this high degree of strategic deviation may also result in firms being unable to fully align with the policy orientation of NEDPs, thereby limiting their performance in substantive green innovation.

**Table 5:** Heterogeneity analysis results.

Variable	(1)	(2)	(3)	(4)	(5)	(6)
**NEDP**	0.134^***^	0.031	0.132^**^	0.037	0.057^*^	0.383^*^
	(0.044)	(0.040)	(0.054)	(0.031)	(0.031)	(0.195)
**Control variable**	YES	YES	YES	YES	YES	YES
**Industry fixation**	YES	YES	YES	YES	YES	YES
**Time fixation**	YES	YES	YES	YES	YES	YES
**City fixation**	YES	YES	YES	YES	YES	YES
**Chow test**	0.008		0.000		0.000	
**_cons**	−0.482	−0.641	0.067^***^	0.057^***^	−0.466	−1.258
	(0.818)	(0.690)	(0.004)	(0.003)	(0.616)	(1.604)
**N**	13341	13300	10408	10590	25066	2615
**R** ^**2**^	0.683	0.703	0.399	0.349	0.669	0.560

**Note:** 1)

* p<0.1,

** p<0.05,

*** p<0.01, are significant at 10%, 5%, and 1% confidence levels, respectively. 2) Robust standard errors, which are clustered at the enterprise level, are shown in parentheses.

#### 5.4.2. Heterogeneity in corporate carbon emission performance.

In Column (3) of Table 5, the estimated coefficient for NEDP is significantly positive at the 5% level, whereas in Column (4), the estimated coefficient for NEDP is positive but not statistically significant. This suggests that national ecological industrial demonstration parks have a more pronounced effect in promoting substantial green innovation among enterprises with higher carbon emissions, whereas the effect is limited for enterprises with lower carbon emissions. This disparity may be attributed to the fact that park policies and regulations are more strictly enforced on high-emission enterprises [53], and these enterprises have a stronger incentive to engage in green innovation due to greater market competition pressure and greater potential for emission reduction. In contrast, low-emission enterprises may exhibit weaker driving forces for green innovation due to limited emission reduction potential, a weaker foundation for technological innovation, or less focused support from the park.

#### 5.4.3. The heterogeneity of resource-based cities.

In Columns (5) and (6) of Table 5, the coefficients for NEDP show a statistically significant positive relationship at the 1% level. Notably, the coefficients for resource-based cities are even higher, suggesting that national ecological industrial demonstration parks have a more pronounced effect in promoting substantial green innovation among enterprises located in these cities. This may be attributed to the fact that resource-based cities often grapple with issues such as monocultural industrial structure and severe environmental pollution. Consequently, the policies and regulations implemented in NEDPs in these cities are likely to be more specific [14]. Such a targeted policy environment helps to incentivize enterprises to increase their investments in green innovation and enhance their environmental protection technologies. Furthermore, resource-based cities tend to exhibit path dependence in their economic development, which may lead to inertia among enterprises in terms of substantial green innovation. However, the establishment of NEDPs has broken through this inertia by introducing advanced environmental protection concepts and technological means, thereby promoting green transformation and innovative development among enterprises.

### 5.5. Mechanism analysis

The preceding theoretical analysis suggests that NEDPs may promote substantive green innovation among enterprises through the pressure effects of heightened government environmental protection attention and increased corporate R&D investment, as well as the incentive effects of government green financial support and the concentration of innovative talent. Following the methodology of Chen et al.(2020) [30], the following model is constructed for analysis:


$$M_{i,l,t}=\alpha_{0}+\alpha_{1}NEDP_{i,l,t}+\sum_{m}\alpha_{m}Controls_{i,l,t}+\lambda_{t}+\mu_{i}+\gamma_{l}+\varepsilon_{i,l,t}$$ (3)

$$GTI_{i,t}=\alpha_{0}+\alpha_{1}NEDP_{i,l,t}+\sum_{m}\alpha_{m}Controls_{i,l,t}+\lambda_{t}+\mu_{i}+\gamma_{l}+\varepsilon_{i,l,t}$$ (4)

*M*_*i,l,t*_ represents four mechanism variables respectively: government environmental protection attention, enterprise research and development investment, government green financial support, and innovative talent aggregation. The other variables and specifications remain the same as in Model (1).

#### 5.5.1. The effect of enhancing government’s focus on environmental protection.

The indicator for government environmental protection attention follows the approach of Bao and Liu (2022) [54], utilizing a textual content dataset of Chinese local governments to extract relevant keywords and quantify them. The results displayed in Column (1) of Table 6 indicate that the coefficient for NEDP is statistically significant and positive at the 1% level, implying that the establishment of NEDPs does indeed foster substantial green innovation. among enterprises by heightening the government’s attention to environmental protection. The possible reason lies in the mandatory norms established by the parks through clear construction standards and quantitative assessments conducted every three years, which enhance local governments’ focus on environmental protection [3]. This not only prompts enterprises within the parks to continuously pursue cleaner and more circular production processes, driving substantive green technological innovation and improving material and energy exchange efficiency, but also encourages enterprises outside the parks to actively strengthen their environmental awareness to secure resource support and meet government green procurement requirements. These enterprises shift from short-term pollution control to long-term pollution prevention, continuously innovating to shape a green image and gain a competitive edge in the market.

**Table 6:** Mechanism analysis results.

Variable	(1)	(2)	(3)	(4)
**NEDP**	0.049^***^	0.529^**^	0.043^***^	0.003^***^
	(0.008)	(0.223)	(0.015)	(0.000)
**Control variable**	YES	YES	YES	YES
**Industry fixation**	YES	YES	YES	YES
**Time fixation**	YES	YES	YES	YES
**City fixation**	YES	YES	YES	YES
**_cons**	0.097	−0.682	−0.712	−6.859^***^
	(1.538)	(0.608)	(0.576)	(1.707)
**N**	8.971^***^	10.001^*^	−3.153^***^	0.152^***^
**R** ^**2**^	(0.102)	(5.312)	(0.188)	(0.006)

**Note:** 1)

* p<0.1,

** p<0.05,

*** p<0.01, are significant at 10%, 5%, and 1% levels, respectively. 2) Robust standard errors are in parentheses, clustered at enterprise level.

#### 5.5.2. The pressure effect of increasing enterprise research and development investment.

Following the methodology employed by Tan et al. (2023) [55], the indicator for R&D investment is calculated as the ratio of R&D expenditure to operating revenue. The findings presented in Column (2) of Table 6 reveal that the coefficient for NEDP is statistically significant and positive at the 5% level, suggesting that national ecological industrial demonstrations do indeed promote substantive green innovation among enterprises by increasing their R&D investment. The potential reason lies in the “innovation compensation effect” triggered by the policy within these parks when the benefits of R&D investment outweigh the exit costs. This effect encourages enterprises within the parks to opt for increasing R&D investment rather than exiting polluted industries, thereby facilitating substantive green innovation. Additionally, the parks foster green knowledge spillovers by constructing green industrial chains. This not only drives compliant innovation among internal enterprises but also inspires external enterprises to actively engage in green technological innovation through collaborative R&D and synchronized technological upgrades.

#### 5.5.3. The incentive effect of government green fiscal support.

Green fiscal policy is measured by the proportion of government expenditure on environmental protection within the public budget expenditure. The results presented in column (3) of Table 6 indicate that the estimated coefficient for NEDP is significantly positive at the 1% level, suggesting that NEDPs can indeed promote substantial green innovation among enterprises by expanding government support through green fiscal measures. The possible reasons for this are as follows: Thirdly, Resource-Based View asserts that firms leverage resources to reduce market risks, expand green markets, and drive technological innovation. NEDPs must adhere to strict environmental protection standards, prompting increased government green investments and economic incentives to encourage enterprises to purchase environmental protection equipment and promote green innovation technologies. The government prioritizes allocation of production factors to low-carbon industries, attract green industries to parks, and expands green innovation entities. Green procurement amplifies fiscal funds’ impact, guiding enterprises to introduce green processes, update green products, and reduce market uncertainty. Green investments also facilitate infrastructure construction for green industries, achieving economies of scale in green technological innovation.

#### 5.5.4. The incentive effect of innovative talent agglomeration.

Drawing inspiration from the approach employed by Cao et al.(2023) [56], we utilize the gravity model to measure urban population mobility. The results displayed in column (4) of Table 6 show that the coefficient for NEDP is statistically significant and positive at the 1% level, suggesting that NEDPs indeed promote substantial green innovation among enterprises by aggregating innovative talent. Possible reasons for this, according to the “voting with one’s feet” theory, innovative talent prefers regions with high – quality environments. By imposing stringent environmental protection standards, NEDPs enhance urban ecology, thus attracting innovative talent. Furthermore, these parks facilitate the transformation of industries towards emerging, modern, and high – end sectors, increasing the diversity of enterprise types and attracting a workforce to support technological innovation. Additionally, the parks’ comprehensive innovation ecosystems and platforms, combined with close collaborations with universities and research institutions, provide innovative talent with abundant R&D resources and entrepreneurial opportunities, fostering the sharing of green knowledge.

## 6. Conclusions and implications

As a pivotal strategy for driving sustainable economic development, corporate green innovation holds significant practical value in exploring whether green location-oriented policies can effectively stimulate micro-enterprises to achieve profound green innovation through a “point-to-area” model. Utilizing China’s National Eco-Industrial Parks as a case study, this research applies a staggered Difference-in-Differences model analysis, along with data sourced from Shanghai and Shenzhen A-share listed companies spanning from 2007 to 2022, to confirm the positive impact of green location-oriented policies on corporate substantial green innovation. The research further reveals that this incentive effect is particularly pronounced in enterprises with low strategic differentiation, low carbon emission performance, and those located in resource-based cities. Mechanistically, NEDPs drive corporate substantial green innovation through multiple effects, including enhancing government attention to environmental protection, increasing pressure on corporate R&D investment, providing green financial support, and attracting the clustering of innovative talents. Drawing from the research findings, the following suggestions are put forth:

To begin with, to fully leverage the role of NEDPs in promoting green innovation among enterprises, the government needs to adopt a two-pronged approach. On the one hand, it should continue to refine location-specific green policies and expand their coverage. It should encourage regions to apply for the establishment of national eco-industrial demonstration parks and incentivize relevant enterprises to proactively adjust their strategic directions to align with regional location-specific green policies through a combination of measures, such as establishing special funds, offering one-time rewards and R&D investment subsidies, implementing tax breaks, and providing low-interest loans. On the other hand, the government should organize multiple parties to formulate substantive green innovation standards for enterprises that encompass green technology R&D, product production, process improvement, and other aspects. Additionally, it should establish a green innovation assessment mechanism to further emphasize the goal of substantive green innovation among enterprises under the location-specific green policies.

Next, the government should continuously increase its environmental focus and compel enterprises to increase substantive innovation investments. It can formulate clear and actionable management regulations for the construction of NEDPs and establish an environmental supervision mechanism. For example, it can link environmental violations to corporate R&D investments, restricting production investments for non-compliant enterprises until they rectify and meet the standards. The government can also utilize intelligent management platforms to monitor corporate carbon emissions in real-time, driving enterprises to proactively pursue clean and circular transformations. By relying on innovation compensation mechanisms, the government can provide financial compensation and tax incentives based on enterprises’ environmental protection investments and green innovation achievements, thereby motivating enterprises to increase R&D investments. Furthermore, it should encourage the establishment of green technology sharing libraries both inside and outside the parks to support joint R&D efforts among enterprises and promote the spillover and sharing of green technologies.

In addition, the government should deepen the incentive effects of green finance and talent agglomeration on substantive green innovation among enterprises. It should expand green investments and establish incentive measures such as green credit, green insurance, green funds, and other specialized environmental protection funds to support green innovation among enterprises. Utilizing government green procurement can also reduce market uncertainty. By introducing “special loans for green talents” and implementing deferred tax policies for technology equity participation, the government can attract talents from other regions to adopt a “migratory bird” residence model. This will concentrate leading talents and technical experts in the field of green innovation, providing diversified talent support and abundant R&D resources for the parks. Simultaneously, the construction of shared laboratories and pilot test bases should be synchronized to form an industry-university-research collaborative network, ultimately creating an efficient cycle of financial capital, innovation elements, and the environmental protection industry to better promote green technological breakthroughs in the parks.

Finally, the precise support strategies of location-specific green policies should be strengthened. Specifically, based on NEDPs, “green technology pilot zones” can be further delineated within cities, and enterprises can be regularly organized to conduct cross-regional technology exchanges and industrial docking activities to learn advanced green technologies and management experiences. At the same time, special subsidies for green innovation should be established, providing direct rewards to enterprises that adopt advanced green technologies and achieve significant energy savings and emission reductions, thereby driving technological green transformations among high-energy-consuming enterprises. For enterprises with low levels of strategic differentiation, the government can draw on the successful model of the Suzhou Industrial Park and encourage enterprises to specialize. The government should formulate special subsidy and tax incentive policies to support enterprises in conducting green technology R&D and energy-saving emission reduction projects, reducing relevant taxes and fees, lowering innovation costs, and stimulating the intrinsic motivation for green specialized development among enterprises. Additionally, a tiered punitive tax policy should be implemented for enterprises with low carbon emission performance, adjusting the tax rate based on their carbon emission intensity and the completion of emission reduction targets. For enterprises with severe carbon emission exceedances and inadequate rectification efforts, the tax collection intensity should be gradually increased.

## Supporting information

S1 Data Regression dataset for substantive green innovation of enterprises. (XLSX)
